# Non–Laboratory-Based Self-Assessment Screening Score for Non-Alcoholic Fatty Liver Disease: Development, Validation and Comparison with Other Scores

**DOI:** 10.1371/journal.pone.0107584

**Published:** 2014-09-12

**Authors:** Yong-ho Lee, Heejung Bang, Young Min Park, Ji Cheol Bae, Byung-Wan Lee, Eun Seok Kang, Bong Soo Cha, Hyun Chul Lee, Beverley Balkau, Won-Young Lee, Dae Jung Kim

**Affiliations:** 1 Department of Internal Medicine, Yonsei University College of Medicine, Seoul, South Korea; 2 Division of Biostatistics, Department of Public Health Sciences, School of Medicine, University of California Davis, Davis, California, United States of America; 3 Department of Family Medicine, National Health Insurance Corporation Ilsan Hospital, Goyang, South Korea; 4 Division of Endocrinology and Metabolism, Department of Internal Medicine, Samsung Medical Center, Sungkyunkwan University School of Medicine, Seoul, South Korea; 5 Inserm, Centre for research in Epidemiology and Population Health (CESP), U1018, Epidemiology of diabetes, obesity and chronic renal disease over the lifecourse, Villejuif, France; 6 University Paris-Sud, UMRS 1018, Villejuif, France; 7 Division of Endocrinology and Metabolism, Department of Internal Medicine, Kangbuk Samsung Hospital, Sungkyunkwan University School of Medicine, Seoul, South Korea; 8 Department of Endocrinology and Metabolism, Ajou University School of Medicine, Suwon, South Korea; 9 Institute on Aging, Ajou University School of Medicine, Suwon, South Korea; Bambino Gesu' Children Hospital, Italy

## Abstract

**Background:**

Non-alcoholic fatty liver disease (NAFLD) is a prevalent and rapidly increasing disease worldwide; however, no widely accepted screening models to assess the risk of NAFLD are available. Therefore, we aimed to develop and validate a self-assessment score for NAFLD in the general population using two independent cohorts.

**Methods:**

The development cohort comprised 15676 subjects (8313 males and 7363 females) who visited the National Health Insurance Service Ilsan Hospital in Korea in 2008–2010. Anthropometric, clinical, and laboratory data were examined during regular health check-ups and fatty liver diagnosed by abdominal ultrasound. Logistic regression analysis was conducted to determine predictors of prevalent NAFLD and to derive risk scores/models. We validated our models and compared them with other existing methods using an external cohort (N = 66868).

**Results:**

The simple self-assessment score consists of age, sex, waist circumference, body mass index, history of diabetes and dyslipidemia, alcohol intake, physical activity and menopause status, which are independently associated with NAFLD, and has a value of 0–15. A cut-off point of ≥8 defined 58% of males and 36% of females as being at high-risk of NAFLD, and yielded a sensitivity of 80% in men (77% in women), a specificity of 67% (81%), a positive predictive value of 72% (63%), a negative predictive value of 76% (89%) and an AUC of 0.82 (0.88). Comparable results were obtained using the validation dataset. The comprehensive NAFLD score, which includes additional laboratory parameters, has enhanced discrimination ability, with an AUC of 0.86 for males and 0.91 for females. Both simple and comprehensive NAFLD scores were significantly increased in subjects with higher fatty liver grades or severity of liver conditions (e.g., simple steatosis, steatohepatitis).

**Conclusions:**

The new non–laboratory-based self-assessment score may be useful for identifying individuals at high-risk of NAFLD. Further studies are warranted to evaluate the utility and feasibility of the scores in various settings.

## Introduction

Non-alcoholic fatty liver disease (NAFLD) is pathologically defined as accumulation of fat, mainly triglycerides, in hepatocytes, with no evidence of significant alcohol consumption or other secondary causes [1], [2]. This includes the entire spectrum of fatty liver conditions, ranging from simple hepatic steatosis through steatohepatitis to cirrhosis. NAFLD is one of the most common metabolic liver disorders, and its incidence is increasing rapidly. The prevalence of NAFLD is between 6.3% and 33% depending on the population [2]–[4], and is expected to rise in the future as the rate of obesity increases, populations become older, and physical activity levels decrease.

NAFLD is associated with serious complications and mortality, and places a large burden on public healthcare systems, as well as patients [5], [6]. It not only impairs health-related quality of life, but is also closely related to metabolic syndrome, dyslipidemia, diabetes, and cardiovascular disease [5], [7], [8]. Subjects with NAFLD demonstrated increased all-cause, cardiovascular and liver-related mortality in general US [9], European [10] and Asian populations [11].

Considering the clinical impact of NAFLD on public health, and its high prevalence, timely screening and detection could be essential to avoid further NAFLD-related morbidity, reduce healthcare costs, and promote early lifestyle interventions that may prevent or delay deterioration of the disease [12]. As NAFLD is usually asymptomatic, it is difficult to predict or determine whether individuals have NAFLD in community settings. NAFLD is diagnosed mostly using imaging modalities such as hepatic ultrasound or computed tomography. These methods are expensive, and can be complicated or inconvenient, and are thus not practical or feasible in the general population. Therefore, establishing a simple screening test or risk assessment tool could be useful not only for identifying individuals at high-risk of NAFLD, but also educating the general public about associated risk factors [13]. A few risk-assessment algorithms have been developed to identify individuals at high-risk of NAFLD [14]–[18]. Most were derived from relatively small samples (*e*.*g*., <600 subjects) and lack external validation, and all models include variables that are less practical or feasible, such as laboratory profiles that require additional blood assays and/or complicated equations that require calculators. These major barriers, which prevent laypersons from using these models, may partly explain why they have not been widely accepted in practice.

Therefore, the aim of our study was to develop and validate a self-assessment score for NAFLD risk in the general population using simple clinical parameters − including demographics, anthropometrics and lifestyle risk factors − to provide a reliable and easy tool usable by laypersons with or without the assistance of a clinician. We also developed a more accurate and comprehensive model that can further account for biochemical parameters when this information is available. Finally, we compared the new algorithm with existing models.

## Materials and Methods

### Ethics statement

The study protocol was approved by the institutional review board of Ilsan Hospital (SU-YON 2013-02). Informed consent requirement for this study was exempted by the institutional review board because researchers only accessed the database for analysis purposes, and personal information was not accessed.

### Data Source and Subjects

The ‘development’ cohort, named the National Health Insurance Service (NHIS) Registry, was established from 18765 individuals aged ≥20 years who visited the NHIS Ilsan Hospital in Korea for comprehensive health examinations between 2008 and 2010, and was used for prediction modeling. Figure S1 illustrates a flow diagram of the study design. Subjects who met the following criteria were excluded based on our protocol: (1) alcohol consumption >140 g/week for males and 70 g/week for females (N = 778); (2) positive serologic markers for hepatitis B (N = 752), hepatitis C virus (N = 130), or human immunodeficiency virus (N = 1); (3) presence of thyroid disease, including hyperthyroidism, hypothyroidism, or thyroid hormone replacement therapy (N = 118); (4) abnormal ultrasonographic liver findings (*i*.*e*., suspected hepatocellular carcinoma, hepatic mass, or signs of *Clonorchis sinensis*) (N = 971); and/or (5) absence of questionnaire data or anthropometric measurements (N = 1135). Ultimately, 15676 subjects (8313 males and 7363 females) were eligible for the analysis.

The ‘external validation’ dataset was obtained from comprehensive health check-up data for Kangbuk Samsung Hospital from 2008, which have been described previously in detail [19]. Briefly, 66868 subjects aged ≥20 years (46896 males and 19972 females) were selected for the validation study after applying the same set of exclusion criteria.

### Data and Measurements

We used demographic and personal and family medical history data, and data on lifestyle/behavioral factors such as smoking and alcohol consumption, physical activity, and anthropometrics. Laboratory parameters were also measured in the morning after overnight fasting for at least 8 h. Subjects previously diagnosed with diabetes by a healthcare professional or taking anti-diabetic drugs based on the health interview survey were classified as having diabetes. Hypertension was defined as diagnosis by a physician or treatment with antihypertensive medication. We defined dyslipidemia according to the National Cholesterol Education Program-Adult Treatment Panel III [20] as a total cholesterol level of ≥200 mg/dL, a triglyceride level of ≥150 mg/dL, a HDL cholesterol level of <45 mg/dL in males or <50 mg/dL in females, a LDL cholesterol level of ≥130 mg/dL, or self-reported use of prescribed cholesterol-lowering medication. Smoking status was categorized as never, ex-, or current smoker on the basis of lifetime exposure to cigarettes. Daily alcohol consumption was quantitated by types of beverages, frequency of drinking, and average amount of alcohol consumed on each occasion, as described previously [21]. After excluding subjects with excessive alcohol intake (as one of the exclusion criteria), alcohol consumption was categorized as non-drinker or current drinker. Exercise status was assessed by self-reported questionnaires that included questions about the duration, frequency, and types of exercise. Regular exercise was then defined as engaging in physical activity for at least 30 min twice or more per week.

In all subjects, abdominal ultrasonography (Sonoline Antares MSC 2704 AB, Siemens Medical Solutions, Issaquah, WA) was performed using a 3.5-MHz transducer by trained radiologists who were blinded to the patients’ clinical and laboratory data. The severity of fatty liver was categorized into three grades–mild, moderate, and severe–based on standard criteria [22]. Then, we finally defined the status of fatty liver as presence *vs*. absence.

### Statistical Analyses

In the development and validation datasets, continuous variables are expressed as the means ± standard deviation (SD), and categorical variables are presented as frequencies with percentages. For model development, we performed multiple logistic regression analyses with NAFLD as the endpoint. We included a list of candidate predictors for NAFLD in an initial regression model, with variables selected based on *P*-values<0.2 in univariate analyses [23]. To create the ‘comprehensive’ model from the initial model, backward elimination was performed until we generated a final model with statistically significant covariates. Then, we further derived a simpler, parsimonious model that could be used by patients for self-assessment with or without input from a clinician. In the ‘simple’ model, laboratory parameters were avoided and continuous variables were categorized using user-friendly cut-off points. We created a weighted scoring system by assigning β coefficients in the final model to integer values, while preserving monotonicity. Of note, in variable selection, categorization, and scoring, clinical and practical judgment as well as statistical significance were utilized [24]. We decided to develop sex-specific models to account for the somewhat different risk factors and cut-off points for different sexes. The goodness of fit of the models was assessed using the Akaike information criterion (AIC), and the discrimination ability by area under the receiver operating characteristic curve (AUC).

Next, we compared our new risk scores with the following screening models for NAFLD using the development and validation datasets: the Fatty Liver Index [14] and NAFLD liver fat score [15] from European populations; the Hepatic steatosis index [16]; and Park’s index for NAFLD [18] from Asian populations. As evaluation measures, we computed sensitivity, specificity, positive predictive value (PPV), negative predictive value (NPV), likelihood ratios (LRs) (positive and negative), the Youden index, and AUC [21], [25], [26]. Imputation was used to handle missing data for fasting insulin (development dataset), menopause status and alcohol consumption (validation dataset). Additionally, we fitted the simple model (derived from the development dataset) to (1) the validation dataset, to assess the consistency of the results, and (2) subjects with excessive alcohol intake (N = 691), to assess the sensitivity/robustness of its discrimination ability. NAFLD fibrosis score [27] was used as a surrogate index for defining poor condition of NAFLD (advanced fibrosis). The formula is: NAFLD fibrosis score = 1.675+0.037×age (years)+0.094×BMI (kg/m^2^)+1.13×IFG/diabetes (yes = 1, no = 0)+0.99×AST/ALT ratio 0.013×platelet (×10^9^/l) 0.66×albumin (g/dl). Jonckheere-Terpstra test was used to conduct trend analysis. Statistical analyses were conducted using SAS version 9.2 (SAS Institute, Cary, NC), SPSS version 20.0 (SPSS, Chicago, IL) and MedCalc (version 13.1).

## Results

### Characteristics of subjects in the development and validation datasets

The characteristics of the study subjects are summarized in Table 1 according to NAFLD status. The prevalences of NAFLD (based on ultrasonographic findings) were 41 and 30% in the development and validation datasets, respectively. The higher prevalence in the development dataset may be explained by the higher mean age. In both datasets, subjects with NAFLD tended to be older and more obese, to exercise less, and to have higher laboratory values for metabolic factors, compared to those without NAFLD. Furthermore, males, those with hypertension or diabetes, and postmenopausal females were more likely to have NAFLD.

**Table 1:** Characteristics of the study subjects.

	Development dataset(N = 15676)		P†	External validationdataset (N = 66868)		P†
	Normal (N = 9221)	NAFLD (N = 6455)		Normal (N = 46896)	NAFLD (N = 19972)	
Age (years)	46.1±11.3	50.5±11.2	<0.001	41.8±8.7	43.7±8.7	<0.001
Sex, M/F(% female)	4054/5167 (56)	4259/2196 (34)	<0.001	21278/25618 (55)	15942/4030 (20)	<0.001
BMI (kg/m^2^)	22.3±2.5	25.9±2.8	<0.001	22.4±2.6	25.9±2.8	<0.001
Waistcircumference (cm)	78.7±7.4	88.6±7.2	<0.001	76.9±7.7	86.8±7.7	<0.001
Fastingglucose (mg/dL)	90.5±13.5	101.2±23.8	<0.001	92.9±12.7	101.6±21.7	<0.001
Uric acid (mg/dL)	4.9±1.2	5.6±1.4	<0.001	5.0±1.3	6.1±1.4	<0.001
Total cholesterol(mg/dL)	186.3±33.0	201.5±37.1	<0.001	190.5±32.0	207.0±34.7	<0.001
Triglycerides(mg/dL)*	89.0±54.3	155.4±110.4	<0.001	101.4±58.2	176.3±106.1	<0.001
HDL cholesterol(mg/dL)	51.0±13.5	42.8±10.6	<0.001	58.1±13.0	48.9±9.8	<0.001
LDL cholesterol(mg/dL)	117.5±29.5	127.6±35.0	<0.001	105.7±27.6	122.7±30.1	<0.001
AST (IU/L)*	22.9±12.7	28.4±14.8	<0.001	22.5±9.4	28.0±12.6	<0.001
ALT (IU/L)*	20.0±15.8	32.2±22.7	<0.001	20.6±13.8	37.1±23.5	<0.001
Hypertension (%)	861 (9)	1489 (23)	<0.001	2081 (4)	2087 (10)	<0.001
Diabetes (%)	258 (3)	692 (11)	<0.001	1027 (2)	1449 (7)	<0.001
Regular exercise (%)	2824 (31)	1843 (29)	0.005	9258 (20)	3357 (17)	<0.001
Smoking history(never/past/current)	5789/1490/1942	3024/1613/1818	<0.001	33601/5192/8103	9858/3895/6219	<0.001
Alcohol consumption(%)	1388 (15)	977 (15)	0.886	NA	NA	NA
Menopause(% of females)	1226 (24)	1219 (56)	<0.001	NA	NA	NA

*Log transformed.

† P values were calculated from t-tests for continuous variables and chi-squared tests for categorical variables, respectively.

M, male; F, female; BMI, body mass index; ALT, alanine aminotransferase; AST, aspartate aminotransferase; NA, not available.

### Development of comprehensive and self-assessment models/scores for NAFLD

Table 2 describes the final−comprehensive and simple−regression models derived from the development dataset. In the comprehensive model, age, alcohol consumption, and regular exercise were significantly associated with NAFLD in males, while diabetes and menopause were significantly associated with NAFLD in females. WC, BMI, and laboratory covariates such as fasting glucose, lipid profiles, uric acid, and liver enzymes were significant predictors, regardless of sex. The comprehensive model yielded an AUC of 0.86 for males and 0.91 for females. The score derived from the comprehensive model (designated the ‘comprehensive score’) ranges from 0 to 100 and can be directly interpreted as the ‘average’ probability of having the disease among persons with similar risk factor profiles.

**Table 2:** Logistic regression analyses of factors related to NAFLD in the development dataset.

	Comprehensive model			Simple model			
Variables		Male (N = 8313)	Female (N = 7363)	Male (N = 8313)	Scoreassigned	Female (N = 7363)	Scoreassigned
		OR (95%CI)	OR (95%CI)	OR (95%CI)		OR (95%CI)	
Age (years)		1.02 (1.01–1.02)	–				
<35				reference		reference	
≥35				2.18 (1.78–2.66)	2	2.42 (1.74–3.38)	2
Waistcircumference (cm)		1.09 (1.08–1.11)	1.05 (1.03–1.06)				
<80(M), 75(F)				reference		reference	
80–90(M), 75–85(F)				3.38 (2.81–4.08)	2	2.96 (2.36–3.70)	1
90–100(M), 85–95(F)				6.86 (5.42–8.68)	3	4.84 (3.71–6.30)	2
≥100(M), 95(F)				12.63 (7.73–20.62)	4	8.07 (5.32–12.24)	3
BMI (kg/m^2^)		1.20 (1.15–1.25)	1.38 (1.32–1.43)				
<23				reference		reference	
23–25				1.78 (1.53–2.07)	1	3.24 (2.75–3.82)	2
25–27				3.33 (2.81–3.95)	2	5.78 (4.71–7.09)	3
≥27				7.31 (5.76–9.27)	3	11.04 (8.41–14.49)	4
Diabetes		–	1.70 (1.20–2.42)*	2.40 (1.94–2.98)	2	3.46 (2.58–4.65)	2
Dyslipidemia		–	–	2.47 (2.12–2.87)	2	2.12 (1.80–2.50)	2
Alcoholconsumption		1.48 (1.28–1.71)	–	1.30 (1.13–1.49)	1	–	
No regular exercise		1.13 (1.01–1.27)^†^	–	1.36 (1.22–1.52)	1	1.17 (1.01–1.36)^†^	1
Menopause		–	1.29 (1.11–1.50)*	–		1.60 (1.40–1.83)	1
Fastingglucose (mg/dL)		1.02 (1.01–1.02)	1.02 (1.01–1.02)				
Triglycerides (mg/dL)‡		2.17 (1.93–2.44)	2.59 (2.23–3.01)				
HDLcholesterol (mg/dL)		0.99 (0.98–0.99)	0.99 (0.98–0.99)				
Uric acid (mg/dL)		1.16 (1.10–1.22)	1.22 (1.13–1.32)				
AST (IU/L)‡		0.51 (0.37–0.70)	0.52 (0.33–0.83)*				
ALT (IU/L)‡		5.11 (4.10–6.37)	3.68 (2.72–4.97)				
AUC		0.86	0.91	0.82		0.88	
Nagelkerke R^2^		0.49	0.54	0.40		0.52	

M, male; F, female; BMI, body mass index; ALT, alanine aminotransferase; AST, aspartate aminotransferase; OR, odds ratios; CI, confidence interval.

*OR for which 0.001≤*P*<0.01; ^†^OR for which 0.01≤*P*<0.05; for all other ORs, *P*<0.001.

‡ Log transformed.

The comprehensive score is calculated as follows: probability (in %) of having NAFLD = 1/(1+exp(−x))×100.

if male,

x = 0.016×age (years)+0.182×BMI (kg/m^2^)+0.089×WC (cm)+0.391×alcohol (yes = 1, no = 0)+0.124×exercise (yes = 0, no = 1)+0.018×fasting glucose (mg/dl)+0.773×log_e_ (triglycerides [mg/dl]) −0.014×HDL cholesterol (mg/dl)+0.145×uric acid (mg/dl) −0.674×log_e_ (AST [IU/l])+1.632×log_e_ (ALT [IU/l]) −21.695.

if female,

x = 0.320×BMI (kg/m^2^)+0.044×WC (cm)+0.533×diabetes (yes = 1, no = 0)+0.016×fasting glucose (mg/dl)+0.951×log_e_ (triglycerides [mg/dl]) −0.015×HDL cholesterol (mg/dl)+0.199×uric acid (mg/dl) −0.645×log_e_ (AST [IU/l])+1.302×log_e_ (ALT [IU/l])+0.255×menopause (yes = 1, no = 0) −19.741.

In the simple model, age, WC, BMI, diabetes, dyslipidemia, and exercise were significant for both sexes (most *P*<0.001). Multiple categories (with scores of 0 to 4) were introduced to capture the risk gradient of obesity measures (BMI and WC), whereas other risk factors were binary. The ‘simple or self-assessment score’ (0–15) was developed from the simple model, where the seven risk factors jointly yielded an AUC of 0.82 for males and 0.88 for females. The AIC and AUC values of the various models are summarized in Table S1.

### External validation

We investigated the diagnostic characteristics of different screening score cut-off points in the development and validation datasets. For the comprehensive score, ≥40 was selected as the cut-off point to define individuals with a high risk of NAFLD as it gives the highest value for the Youden index (data not shown). For the simple score, we selected a cut-off point of ≥8, which yielded sensitivities of 75% and 68%, specificities of 71% and 85%, and AUC values of 0.80 and 0.85 in males and females, respectively (Table 3). The comprehensive score and simple score (in females) yielded the highest overall test accuracy (reflected by the Youden index) and the largest AUC in both datasets, while the performance of the simple score (in males) was comparable in performance of the other risk models. Loss of accuracy and discrimination with the simple score was minimal, despite it not relying on difficult health information or formulae. Comparison analysis of AUC among screening models in the validation dataset showed that the performance of comprehensive score and Fatty liver index were superior to those of other models (Table S2).

**Table 3:** Performance of NAFLD screening scores: development and external validation datasets.

Method, by Dataset	High risk(%)	Sensitivity	Specificity	PPV	NPV	Positive	Negative	Youden	AUC*	RequiresClinician’sInput?
						LR	LR	Index		
**Development** **dataset (N = 15676)**										
Comprehensive score (male) ≥40	43	69	84	82	72	4.46	0.36	54	0.86	Yes
Comprehensive score (female) ≥40	29	72	89	73	88	6.50	0.31	61	0.90	Yes
Simple score (male) ≥8	58	80	67	72	76	2.41	0.29	47	0.82	No
Simple score (female) ≥8	36	77	81	63	89	4.09	0.28	58	0.87	No
Park’s index*	41	72	81	73	80	3.84	0.35	53	0.83	Yes
Hepatic steatosis index*	20	41	95	85	70	7.91	0.62	36	0.85	Yes
Fatty liver index*	12	26	98	89	65	11.09	0.76	23	0.86	Yes
NAFLD liver fat score* ^,^ ‡	19	37	94	81	68	6.26	0.67	31	0.77	Yes
**External validation dataset (N = 66868)**										
Comprehensive score (male)† ≥40	37	67	85	76	77	4.32	0.39	52	0.85	Yes
Comprehensive score (female)† ≥40	20	71	88	49	95	6.13	0.33	59	0.89	Yes
Simple score (male)† ≥8	49	75	71	66	79	2.53	0.36	45	0.80	No
Simple score (female)† ≥8	22	68	85	42	94	4.57	0.38	54	0.85	No
Park’s index*	38	76	78	59	89	3.44	0.30	54	0.84	Yes
Hepatic steatosis index*	21	52	92	74	82	6.56	0.52	44	0.86	Yes
Fatty liver index*	11	30	97	82	76	10.77	0.72	27	0.87	Yes
NAFLD liver fat score* ^,^ ‡	16	40	94	75	79	6.96	0.63	35	0.82	Yes

*The definition of the above risk models for NAFLD.

Park’s index for NAFLD: index ≥3, in which Park’s index = (ALT/AST ratio >1.5)×1+(γ-glutamyl-transferase >50)×1+(triglycerides >150)×1+(23≤ BMI <25)×2+(25≤ BMI)×3.

Hepatic steatosis index (HSI): HSI >36 for high-risk and <30 for no NAFLD, in which HSI = 8×ALT/AST ratio+BMI (+2, if diabetes;+2, if female).

Fatty liver index (FLI): FLI ≥60 for high-risk and <30 for no NAFLD, in which FLI = 1/(1+exp(−x))×100, where x = 0.953×log_e_ (triglycerides)+0.139×BMI+0.718×log_e_ (γ-glutamyl-transferase)+0.053×WC −15.745.

NAFLD liver fat score: score ≥−0.640, in which NAFLD liver fat score = −2.89+1.18×metabolic syndrome+0.45×diabetes+0.15×fasting insulin+0.04×AST+0.94×AST/ALT ratio.

† After imputing the missing data for alcohol consumption and menopausal status.

‡ After imputing the missing data of fasting insulin levels.

When our simple model was refitted to the validation dataset, similar results were obtained: all of the risk factors were statistically significant, and the direction and magnitude of the associations were comparable, with an AUC of 0.80 in males and 0.85 in females (Table S3).

**Figure 1:**
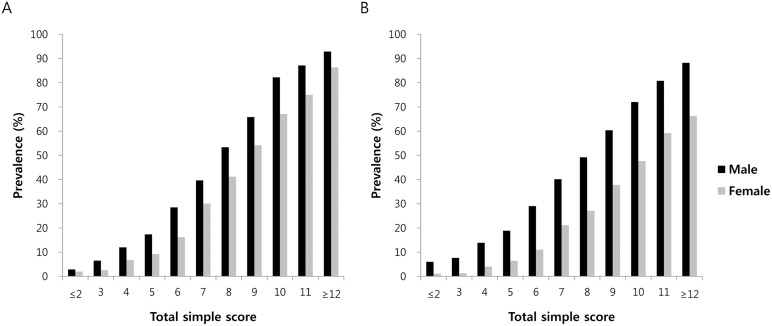
Estimated prevalence of NAFLD according to screening score: development and external validation datasets. A, development dataset (N = 15676); B, external validation dataset (N = 66868).

**Figure 2:**
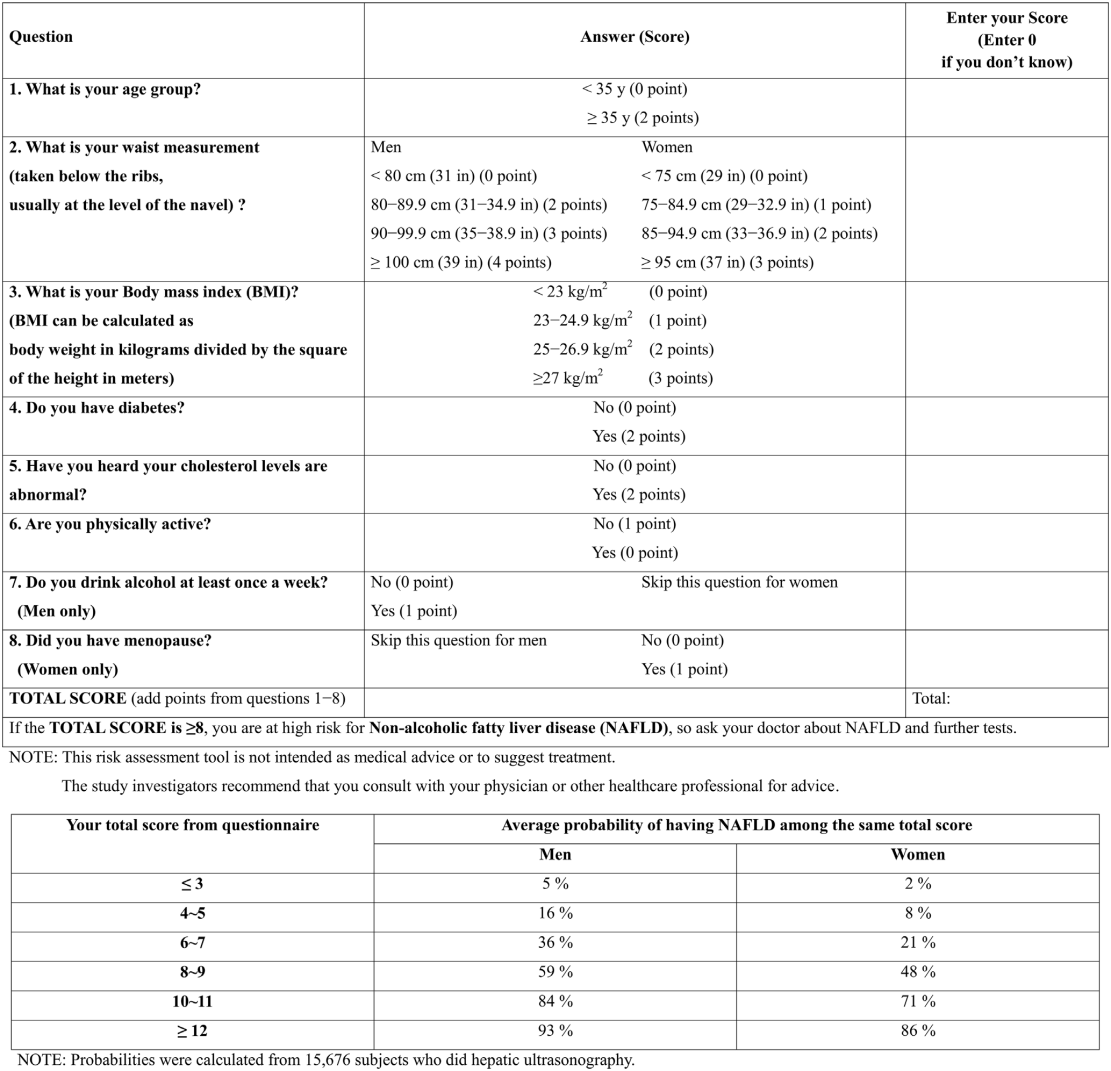
Sample self-assessment screening questionnaire.

Figure 1 shows the prevalence of NAFLD according to total simple score for each sex group in the development and validation datasets. The prevalence of NAFLD increased as the risk scores increased. Figure 2 provides a sample questionnaire for the risk assessment of NAFLD that may be used by laypersons, as well as healthcare providers.

### Ancillary analyses

As a sensitivity analysis, we applied the simple score to the subjects with excessive alcohol consumption (N = 691) who were initially excluded from the analysis. The simple score yielded similar AUCs (0.82 for males and 0.87 for females), suggesting that the discriminatory ability was preserved, even in a specific population highly susceptible to alcoholic fatty liver.

**Figure 3:**
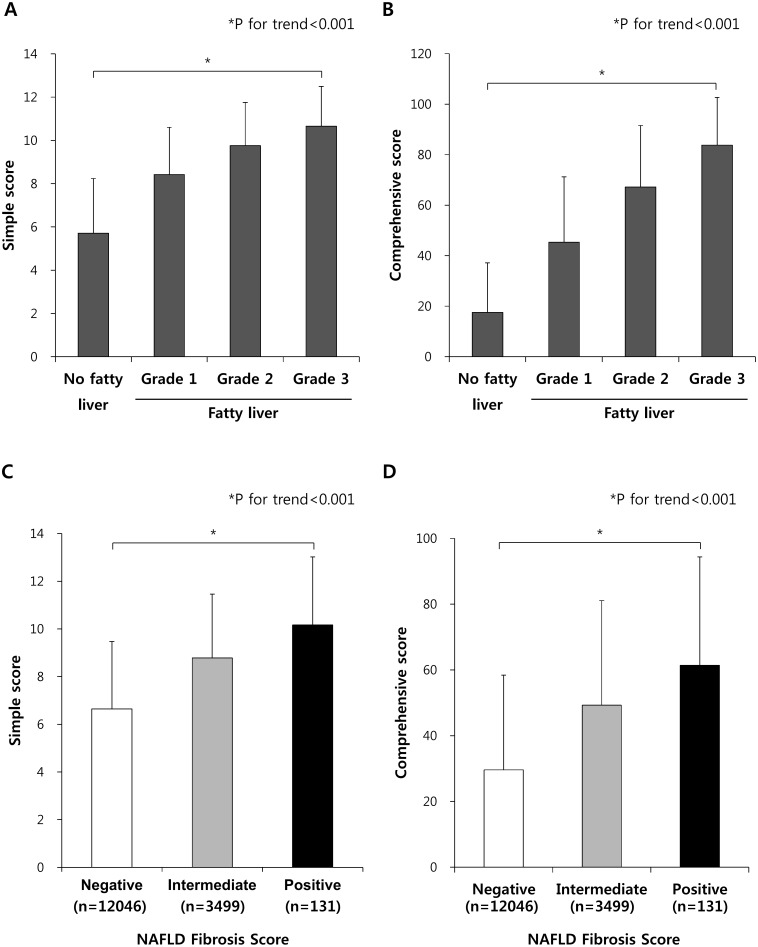
Average scores for the simple and comprehensive models according to fatty liver grade determined by hepatic ultrasound or NAFLD fibrosis score. Average scores for A) the simple model or B) the comprehensive model according to fatty liver grade determined by hepatic ultrasound. All P values of comparison between any groups are <0.001. *P for trend are <0.001. Average scores for C) the simple model or D) the comprehensive model according to the fatty liver conditions defined by NAFLD fibrosis score. Subjects with negative results by NAFLD fibrosis score (N = 12046) could be excluded from having advanced fibrosis and subjects with positive results of NAFLD fibrosis score (N = 131) are highly likely to have advanced fibrosis. All P values of comparison between any groups are <0.001. *P for trend are <0.001. Data are shown as mean with SD.

The simple and comprehensive scores were gradually increased in subjects with higher fatty liver grades (determined by hepatic ultrasound) (all *P*<0.001; Figure 3A and B). Furthermore, subjects categorized as having advanced fibrosis based on NAFLD fibrosis score [27] showed significantly higher simple and comprehensive scores compared to other subjects with negative or intermediate results from NAFLD fibrosis which denotes less likely to have advanced fibrosis in their liver. (all *P*<0.001; Figure 3C and D). These findings indicate that the present scores can reflect the severity of fatty liver and be applied to discriminate advanced stage of non-alcoholic steatohepatitis (NASH) from simple steatosis.

## Discussion

We developed and validated models that could identify subjects at high-risk of NAFLD. The simple model is based on demographic, clinical, and anthropometric variables–age, WC, BMI, diabetes, dyslipidemia, exercise, alcohol intake, and menopause status–while the comprehensive model additionally includes laboratory parameters such as lipid and liver enzyme profiles. Newly developed NAFLD screening models/scores may serve as a doctor–patient communication tool and an initial step to identify high-risk subjects, who can be referred for further blood assays or imaging tests such as ultrasonography, possibly in conjunction with preventive measures or early interventions to manage NAFLD. Depending on the availability of health-related information and targeted accuracy, either or both models may be used.

NAFLD is regarded not only as a common disease in Western societies but also as an emerging problem in many Asian countries [28]. That the prevalence of NAFLD can further increase as the number of obese people in Asia increases is supported by the findings that 65 and 85% of subjects with a BMI of 30–40 and ≥40 kg/m^2^, respectively, had NAFLD [29]. Despite its high prevalence and potential impact, recent studies highlighted that, even among high-risk patients, 87% of people did not know they had NAFLD [30] and 51% of healthy potential liver donors were incidentally confirmed as having NAFLD by liver biopsy [31], indicating that alarming proportions of patients with NAFLD are unaware of their illness and are undiagnosed. Therefore, a simple risk score that can efficiently and effectively screen high-risk individuals for NAFLD in communities, as well as in clinical settings, could help improve personal and population health, and public awareness and education about this less known disease. Notably, even in developed countries such as Hong Kong, 83% of the general population had never heard of NAFLD and 78% of respondents who understood the term had a misconception about this common condition [13]. We speculate that this is also a common phenomenon in other settings.

To date, studies have focused on searching for novel biomarkers or developing models that can predict progression of NAFLD to NASH [27], [32]. Although NASH is a more serious liver disorder that may progress to cirrhosis, early detection of mild forms of NAFLD, such as simple steatosis, is also an important and promising field from a public health perspective. As fatty liver is a more prevalent but reversible disease, identification of individuals at high-risk of NAFLD through proper risk assessment and subsequent lifestyle modification may restore their hepatic condition and prevent progression to NASH or other related morbidities.

Our study has several distinguishable features. First, to our knowledge, the model was developed based on the largest general population with hepatic ultrasound-defined NAFLD. Many studies examined relatively small numbers of subjects [14], [15], [33] or used surrogate markers such as liver enzymes to predict NAFLD [34]; however, a significant number of patients with NAFLD have normal liver enzyme levels. Second, our simple score is easy to use and is based on readily available variables. Thus, laypersons can calculate and learn about their own risk–with or without help from healthcare providers–and can initiate a discussion with their physician if necessary. Third, the score includes modifiable risk factors such as obesity (defined by WC and BMI), exercise, and alcohol consumption, so users can learn about important risk factors and could be motivated to change or improve their lifestyle/health habits. For example, if subjects at high-risk of NAFLD reduce their body weight, start exercising regularly, and abstain from drinking, their risk scores could decrease. Lastly, the sensitivity analysis indicated that the simple score is well applicable to predict hepatic steatosis in subjects with heavy drinking as well. This may be explained by the findings that fatty liver is more strongly affected by obesity than by excessive alcohol consumption [35].

Of note, our scores are sex-specific, unlike in previous models. WC, which reflects central obesity, had a stronger association with NAFLD in males than in females, while BMI, which is an index of overall obesity, showed a stronger association with NAFLD in females compared to males in our two independent datasets. This observation may be due to male subjects having more visceral adipose tissue than females, and suggest that males may be more susceptible to visceral fat deposition, which leads to accumulation of fat in the liver [36]. In females, the OR was dramatically increased in subjects aged over 50 years. This increase was offset by adjustment for menopause status in the logistic model, indicating that menopause may be more influential than age for NAFLD in females. This finding is supported by epidemiologic and experimental evidence that insulin resistance and visceral fat levels are significantly increased in postmenopausal females [37], and that estrogen protects against hepatic steatosis [38]. Among biochemical variables, ALT and AST made the greatest contribution to the prediction of NAFLD, followed by triglycerides, consistent with previous findings [14]–[16], [18]. In addition, one of the components of the comprehensive model was serum uric acid level, which has been proposed to be a risk factor for NAFLD [39].

The present study has some potential limitations, which could be addressed by future investigations. First, diagnosis of NAFLD by ultrasonography could underestimate the actual prevalence of NAFLD in this population. Second, as this risk score was derived from a cross-sectional study, its use for the prediction of future development of NAFLD may be limited. However, cross-sectional data are well suited for screening for prevalent, undiagnosed cases, which generally precedes the prediction of incident, new cases. Third, the models/scores were derived from the general population of an Asian ethnic group, which may limit their generalizability and applicability to non-Asian or other Asian populations. Notably, considering the differences in the definitions of obesity between Western and Asian countries, cut-off points for factors related to obesity (*e*.*g*., WC and BMI) will be subject to modifications depending on the population, or new models may be warranted [40].

## Conclusions

The present results demonstrate that new screening scores for NAFLD performed well compared to existing models, and has some notable advantages (*e*.*g*., no laboratory tests required, self-assessment). Our simple self-assessment score for NAFLD risk could be useful for both primary care practitioners and laypersons as a screening and counseling tool. Future research is warranted to verify the effectiveness, usefulness, and feasibility of our models in various practical settings, and potentially to revise or adapt them for other populations.

## Supporting Information

Figure S1 **Flow diagram of subjects inclusion and exclusion in the development and validation cohorts.**
(DOCX)

Table S1 Goodness of fit of sequential models in the development dataset.
(DOCX)

Table S2 Comparison analysis of area under the curves among NAFLD screening scores.
(DOCX)

Table S3 Final regression model fitted to the external validation dataset.
(DOCX)
