# Supplementary material for: Non–Laboratory-Based Self-Assessment Screening Score for Non-Alcoholic Fatty Liver Disease: Development, Validation and Comparison with Other Scores
Source: PLoS One. 2014 Sep 12;9(9):e107584. doi: 10.1371/journal.pone.0107584 (PMC4162644; doi:10.1371/journal.pone.0107584)

**Figure S1.** Flow diagram of subjects inclusion and exclusion in the development and validation cohort


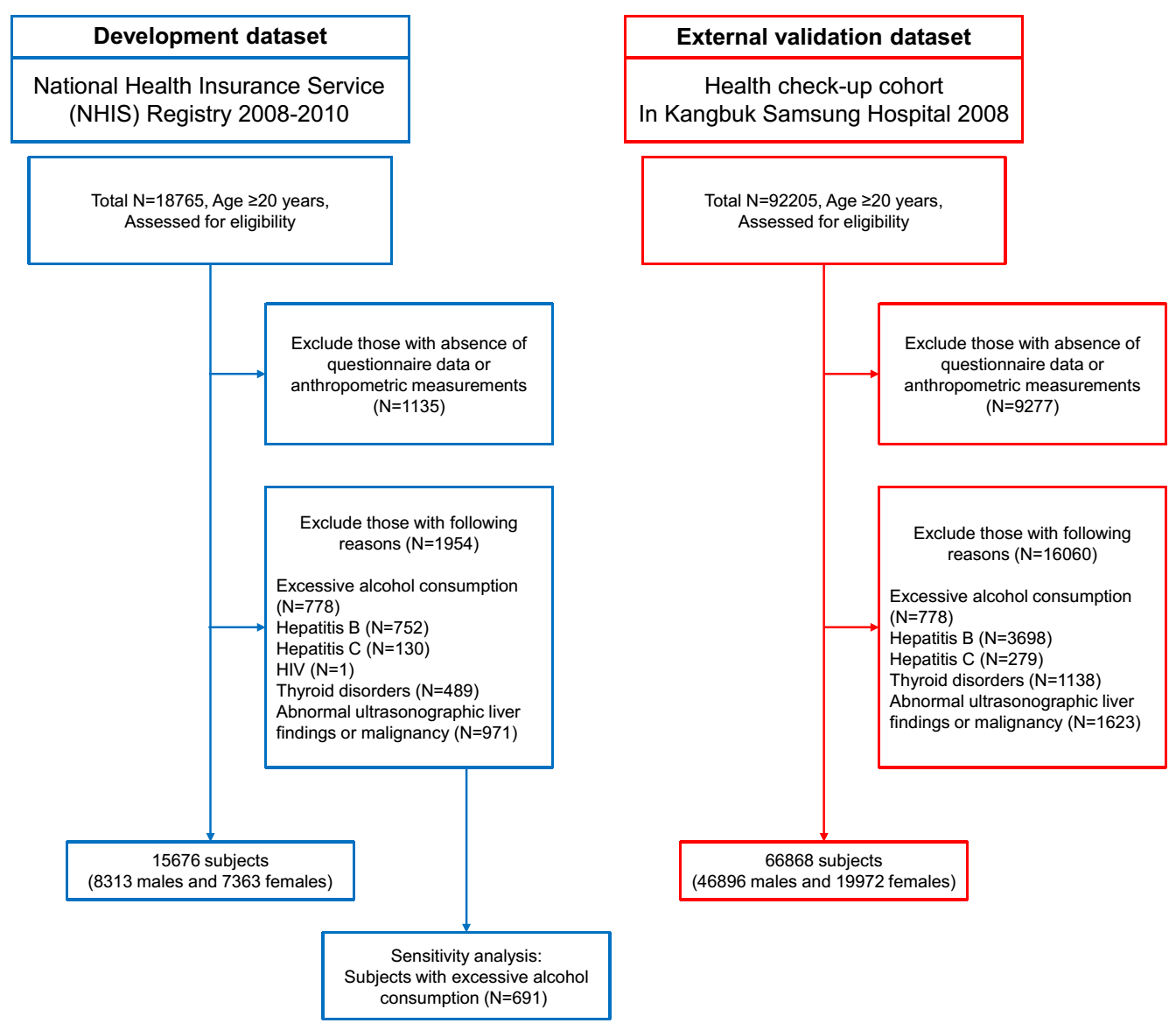

Supplement: Figure S1 — Flow diagram of subjects inclusion and exclusion in the development and validation cohorts. (DOCX) [file pone.0107584.s001.docx]
