# Supplementary material for: Non–Laboratory-Based Self-Assessment Screening Score for Non-Alcoholic Fatty Liver Disease: Development, Validation and Comparison with Other Scores
Source: PLoS One. 2014 Sep 12;9(9):e107584. doi: 10.1371/journal.pone.0107584 (PMC4162644; doi:10.1371/journal.pone.0107584)
Supplement: Table S1 — Goodness of fit of sequential models in the development dataset. (DOCX) [file pone.0107584.s002.docx]

**Table S1. Goodness of fit of sequential models in the development dataset.**

| Variables included in risk models | Males (N = 8313) | |
| --- | --- | --- |
|  | AIC^*^ | AUC^†^ |
| Intercept only | 11521 |  |
| Age | 11417 | 0.532 |
| Age + BMI | 9196 | 0.784 |
| Age + BMI + WC | 8845 | 0.806 |
| Age + BMI + WC + DM | 8784 | 0.810 |
| Age + BMI + WC + DM + dyslipidemia | 8635 | 0.818 |
| Age + BMI + WC + DM + dyslipidemia + exercise | 8609 | 0.821 |
| **Age + BMI + WC + DM + dyslipidemia + exercise + drink** | **8597** | **0.822** |
| Age + BMI + WC + DM + dyslipidemia + exercise + drink + HTN | 8598 | 0.822 |
| Age + BMI + WC + DM + dyslipidemia + exercise + drink + smoking | 8601 | 0.822 |
|  |  |  |
| Variables included in risk models | Females (N = 7363) | |
|  | AIC^*^ | AUC^†^ |
| Intercept only | 8976 |  |
| Age | 7216 | 0.558 |
| Age + BMI | 6354 | 0.843 |
| Age + BMI + WC | 6169 | 0.858 |
| Age + BMI + WC + DM | 6072 | 0.864 |
| Age + BMI + WC + DM + dyslipidemia | 5976 | 0.869 |
| Age + BMI + WC + DM + dyslipidemia + menopause | 5947 | 0.876 |
| **Age + BMI + WC + DM + dyslipidemia + menopause + exercise** | **5944** | **0.877** |
| Age + BMI + WC + DM + dyslipidemia + menopause + exercise + drink | 5975 | 0.877 |
| Age + BMI + WC + DM + dyslipidemia + menopause + exercise + smoking | 5947 | 0.877 |
| Age + BMI + WC + DM + dyslipidemia + menopause + exercise + drink + smoking +HTN | 5940 | 0.877 |

^*^Lower AIC values (Akaike information criterion) suggest a better model fit.

^†^Higher AUC values (area under the receiver operating characteristic curve) indicate better discrimination ability.

BMI, body mass index; WC, waist circumference; DM, diabetes; HTN, hypertension
