# Supplementary material for: Non–Laboratory-Based Self-Assessment Screening Score for Non-Alcoholic Fatty Liver Disease: Development, Validation and Comparison with Other Scores
Source: PLoS One. 2014 Sep 12;9(9):e107584. doi: 10.1371/journal.pone.0107584 (PMC4162644; doi:10.1371/journal.pone.0107584)
Supplement: Table S2 — Comparison analysis of area under the curves among NAFLD screening scores. (DOCX) [file pone.0107584.s003.docx]

**Table S2. Comparison analysis of area under the curves among NAFLD screening scores.**

|  | Development dataset (N=15676)^*^ | | | |  | External validation dataset (N=66868)^†^ | | | |
| --- | --- | --- | --- | --- | --- | --- | --- | --- | --- |
|  | Comprehensive score | | Simple score | |  | Comprehensive score | | Simple score | |
| Compared to | Difference between AUC^‡^ | P | Difference between AUC^‡^ | P |  | Difference between AUC^‡^ | P | Difference between AUC^‡^ | P |
| Park’s index | 0.0423 | <0.001 | 0.0003 | 0.917 |  | 0.0319 | <0.001 | -0.0031 | 0.009 |
| Hepatic steatosis index | 0.0418 | <0.001 | 0.0008 | 0.754 |  | 0.0151 | <0.001 | -0.0199 | <0.001 |
| Fatty liver index | 0.0154 | <0.001 | -0.0272 | <0.001 |  | 0.00004 | 0.966 | -0.0350 | <0.001 |
| NAFLD liver fat score | 0.1000 | <0.001 | 0.0577 | <0.001 |  | 0.0517 | <0.001 | 0.0166 | <0.001 |

^*^AUC of comprehensive score and simple score in the development dataset are 0.873 and 0.831, respectively.

^†^AUC of comprehensive score and simple score in the external validation dataset are 0.871 and 0.836, respectively.

^‡^Difference between AUC = AUC of comprehensive or simple score - AUC of an indicated model in each column.
