# Supplementary material for: Non–Laboratory-Based Self-Assessment Screening Score for Non-Alcoholic Fatty Liver Disease: Development, Validation and Comparison with Other Scores
Source: PLoS One. 2014 Sep 12;9(9):e107584. doi: 10.1371/journal.pone.0107584 (PMC4162644; doi:10.1371/journal.pone.0107584)
Supplement: Table S3 — Final regression model fitted to the external validation dataset. (DOCX) [file pone.0107584.s004.docx]

**Table S3. Final regression model fitted to the external validation dataset.**

| Variables | Simple model | | | | | | |
| --- | --- | --- | --- | --- | --- | --- | --- |
|  | Male (N=37220) | | |  | Female (N=29648) | | |
|  | OR | 95%CI | |  | OR | 95%CI | |
| Age, y |  |  |  |  |  |  |  |
| <35 | reference | | |  | reference | | |
| ≥35 | **1.33** | 1.24 | 1.43 |  | **2.06** | 1.79 | 2.38 |
| Waist circumference,(cm) |  |  |  |  |  |  |  |
| <80(M), 75(F) | reference | | |  | reference | | |
| 80-90(M), 75-85(F) | **2.26** | 2.10 | 2.43 |  | **1.99** | 1.78 | 2.23 |
| 90-100(M), 85-95(F) | **3.73** | 3.34 | 4.17 |  | **2.50** | 2.15 | 2.90 |
| ≥100(M), 95(F) | **7.89** | 6.19 | 10.05 |  | **4.39** | 3.45 | 5.59 |
| BMI (kg/m^2^) |  |  |  |  |  |  |  |
| <23 | reference | | |  | reference | | |
| 23-25 | **1.94** | 1.80 | 2.10 |  | **3.05** | 2.74 | 3.40 |
| 25-27 | **3.39** | 3.11 | 3.70 |  | **5.24** | 4.60 | 5.97 |
| ≥27 | **6.08** | 5.41 | 6.82 |  | **10.25** | 8.76 | 11.99 |
| Diabetes |  |  |  |  |  |  |  |
| No | reference | | |  | reference | | |
| Yes | **1.76** | 1.58 | 1.97 |  | **5.94** | 4.85 | 7.28 |
| Regular exercise |  |  |  |  |  |  |  |
| Yes | reference | | |  | reference | | |
| No | **1.49** | 1.40 | 1.59 |  | **1.14^*^** | 1.04 | 1.25 |
| Dyslipidemia |  |  |  |  |  |  |  |
| No | reference | | |  | reference | | |
| Yes | **2.65** | 2.50 | 2.81 |  | **2.75** | 2.51 | 3.01 |

Area under the receiver-operating characteristic curve = 0.80 in males and 0.85 in females.

M, male; F, female; BMI, body mass index; OR, odds ratios; CI, confidence interval

^*^*P*=0.007 (*P*<0.001 for all other ORs).

The alcohol consumption and menopause variables were omitted due to the unavailability of data for them in the validation dataset.
